# Evaluation of Biopolymer Materials and Synthesis Techniques to Develop a Rod-Shaped Biopolymer Surrogate for *Legionella pneumophila*

**DOI:** 10.3390/polym14132571

**Published:** 2022-06-24

**Authors:** Sujani Ariyadasa, Weiam Daear, Gayan Abeysekera, Craig Billington, Conan Fee, Elmar Prenner, Liping Pang

**Affiliations:** 1 Institute of Environmental Science and Research, P.O. Box 29181, Christchurch 8540, New Zealand; gayan.abeysekera@canterbury.ac.nz (G.A.); craig.billington@esr.cri.nz (C.B.); liping.pang@esr.cri.nz (L.P.); 2 School of Biological Sciences, University of Canterbury, Private Bag 4800, Christchurch 8041, New Zealand; 3 Department of Biological Sciences, University of Calgary, Calgary, AB T2N 1N4, Canada; wtdaear@ucalgary.ca (W.D.); eprenner@ucalgary.ca (E.P.); 4 School of Product Design, University of Canterbury, Private Bag 4800, Christchurch 8041, New Zealand; conan.fee@canterbury.ac.nz; 5 Biomolecular Interaction Centre, University of Canterbury, Private Bag 4800, Christchurch 8041, New Zealand

**Keywords:** biopolymer, microparticles, synthesis, biodegradable, biocompatible, surrogate, DNA-loaded, *L. pneumophila*

## Abstract

Biopolymer microparticles have been developed for applications that require biocompatibility and biodegradability, such as drug delivery. In this study, we assessed the production of microparticles using carnauba wax, κ-carrageenan, alginate, and poly (lactic-co-glycolic acid) (PLGA) with the aim of developing a novel, DNA-tracer-loaded, biopolymer surrogate with a size, shape, surface charge, and relative hydrophobicity similar to stationary-phase *Legionella pneumophila* to mimic the bacteria’s mobility and persistence in engineered water systems. We found that the type and concentration of biopolymer, reaction conditions, and synthesis methods affected the morphology, surface charge, relative hydrophobicity, and DNA tracer loading efficiency of the biopolymer microparticles produced. Carnauba wax, κ-carrageenan, and alginate (Protanal^®^, and low and medium viscosity) produced highly polydisperse microspheres. In contrast, PLGA and alginate-CaCO_3_ produced uniform microspheres and rod-shaped microparticles, respectively, with high DNA tracer loading efficiencies (PLGA 70% and alginate-CaCO_3_ 95.2 ± 5.7%) and high reproducibilities. Their synthesis reproducibility was relatively high. The relative hydrophobicity of PLGA microspheres closely matched the cell surface hydrophobicity of *L. pneumophila* but not the bacterial morphology, whereas the polyelectrolyte layer-by-layer assembly was required to enhance the relative hydrophobicity of alginate-CaCO_3_ microparticles. Following this surface modification, alginate-CaCO_3_ microparticles represented the best match to *L. pneumophila* in size, morphology, surface charge, and relative hydrophobicity. This new biopolymer surrogate has the potential to be used as a mimic to study the mobility and persistence of *L. pneumophila* in water systems where the use of the pathogen is impractical and unsafe.

## 1. Introduction

*Legionella pneumophila* (*L. pneumophila*) is an opportunistic premise plumbing pathogen that causes Legionnaires’ disease, a potentially life-threatening pneumonia, particularly in immunocompromised and elderly individuals [1]. The bacteria persist in EWS biofilms in association with other microorganisms and are released into the bulk water upon biofilm maturation, leading to a risk of spread of Legionnaires’ disease. Legionnaires’ disease outbreaks have been reported worldwide, resulting in a significant cost to public health [1]. The disease incidence is thought to have increased the post-COVID temporary closure of industrial buildings due to the stagnation of water [2]. Despite this, the mobility and persistence of the bacteria in EWSs remain poorly studied, mainly due to the lack of a poor indicator or surrogate.

The study of transportation, persistence, and attenuation of pathogens in engineered water systems (EWSs) is essential in understanding the fundamental mechanisms of waterborne disease outbreaks, including Legionnaires’ disease. However, in most circumstances, field studies or environmental applications of waterborne pathogens such as *L. pneumophila* are not possible, due to the health risk of exposure and contamination [3]. Therefore, surrogates are often used to model the mobility and persistence of waterborne pathogens [4]. As such, the selection of a representative, safe surrogate is necessary to prevent the over or under-representation of pathogen behavior in water systems [3]. Bacteriophages, fecal indicator bacteria *E. coli*, bacterial endospores, and fluorescently labeled polystyrene microspheres have been often used as surrogates for bacterial, protozoan, and viral pathogens in water applications [5,6]. However, the dissimilarities between pathogen and surrogate hydrophobicity, surface charge, size, and morphology result in significant differences in their transport behaviors, often by a few orders of magnitude [5,6]. For example, previous studies conducted using *E. coli* [7,8] as a surrogate for *L. pneumophila* have indicated that the former is a poor model for *L. pneumophila* due to the differences in their cell surface hydrophobicity. It is known that the transportation and attenuation of both bacteria and surrogate microparticles are greatly influenced by their size, shape, surface charge, and hydrophobicity [9].

The surface properties of synthetic surrogates have been chemically modified to match the cell surface charge and hydrophobicity of the pathogens to facilitate more accurate predictions of pathogen transport. For example, in a recent study, Pang et al. [10] used protein-coated, DNA-labeled silica nanospheres to mimic the size, surface charge, and hydrophobicity of rotavirus. Similarly, in another study, fluorescent carboxylated polystyrene microspheres were coated with glycoprotein to mimic the surface charge and surface macromolecule of *Cryptosporidium parvum* [11]. The outcome of these studies showed that the similarities in surface properties of surrogates and the pathogen resulted in more representative models. However, applications involving eco-sensitive environments require surrogates constructed using eco-friendly, biocompatible, biodegradable material.

Biopolymers have emerged as a new class of alternative materials to synthetic polymers owing to their biocompatible and biodegradable nature, easy accessibility and processability, low density, adjustable physicochemical properties, and cost-effectiveness [12]. A plethora of methods are available in the literature to prepare biopolymer microparticles with modulated and tunable properties. Biopolymer-based applications are becoming increasingly popular, particularly in the pharmaceutical, biomedical, packaging, agricultural, and environmental engineering applications [13], where biodegradable and biocompatible properties are sought.

To address the need for representative, safe, and detection-sensitive *L. pneumophila* mimics for use in water environments, we focused on the development of a biocompatible, biodegradable surrogate for the bacteria. We evaluated the production of *L. pneumophila* surrogate biopolymer microparticles using a range of biopolymers identified as candidates for the surrogate synthesis process through a review of the literature [14]. The main criteria for biopolymer selection were solubility in water at ambient temperature, overall surface charge, availability of facile methods for microparticle synthesis without the requirement of specialized equipment or hazardous chemicals for potential future upscaling and environmental application, and the availability of functional groups for surface modifications. In addition, the mimics needed to incorporate short DNA tracer sequences to enable their tracking in the environment [15]. Synthesis techniques were evaluated in the laboratory to obtain a rod-shaped biopolymer surrogate with a size, surface charge, and relative hydrophobicity similar to stationary-phase *L. pneumophila*. An accompanying paper [16] describes the testing of this biopolymer surrogate in a model biofilm engineered water system.

## 2. Materials and Methods

Most materials used in microparticle synthesis were purchased from Sigma-Aldrich, St. Louis, MO, USA. These included carnauba wax, κ-carrageenan, poly (lactic-co-glycolic acid) (PLGA), dichloromethane, polyvinyl alcohol (PVA), sodium alginate, sodium carbonate (Na_2_CO_3_), calcium chloride (CaCl_2_), poly-L-lysine hydrobromide (PLL, molecular weight 30,000–70,000), poly-L-glutamic acid sodium salt (PGA, molecular weight 50,000–100,000), poly (L-lactide-co-glycolide) (PLGA, lactide:glycolide ratio = 10:90 ± 5), Span 80, β-cyclodextrin, isoamyl alcohol (≥98%, reagent grade), and dimethyl sulfoxide. Canola oil was obtained from Pams, Auckland, New Zealand.

### 2.1. Microparticle Synthesis and Characterization

**Carnauba wax microparticles**: Carnauba wax microparticles were synthesized by adapting the melt dispersion technique of Milanovic et al. [17]. Briefly, 1% (*w/v*) carnauba wax solution was prepared by gradually adding wax flakes into distilled water heated to 95 °C in a temperature-regulated water bath. Once the wax flakes were melted completely, the mixture was homogenized (Omni tissue homogenizer TH115-K) at 5000 rpm for 8 min and cooled down to 4 °C for the formation and solidification of carnauba wax particles, respectively. Particles were recovered by centrifugation at 8500 rpm for 20 min. Synthesis was conducted at a wax mass fraction of 1%. Homogenization time and speed were increased to 8 min and 5000 rpm, respectively, as opposed to 4 min and 1000 rpm used in the original study, in order to form small microspheres with a narrow size distribution range.

**κ-Carrageenan microparticles**: κ-carrageenan microparticles were prepared by modification of the method of Ellis and Jacquier [18]. Briefly, 2.5 g of κ-carrageenan was emulsified in 50 mL of canola oil heated to 68 °C by homogenizing at 16,250 rpm for 20 min (Omni tissue homogenizer TH115-K). The emulsion was cooled to 10 °C on an ice bath (while homogenizing at 5000 rpm) and centrifuged at 300 rpm for 15 min to collect the particles. Particles were washed 5 times in 100 mM KCl to remove the oil.

**PLGA microparticles**: The water-in-oil-in-water double emulsion solvent evaporation method described by Ando et al. [19] was modified and used to synthesize PLGA microparticles containing a loaded DNA tracer. For microparticle preparation, 1.75 mL of dichloromethane containing 50 mg of PLGA was sonicated for 10 s at room temperature and immersed in liquid nitrogen. An amount of 12.5 mL of 5% PVA (4 °C) was then added and homogenized at 8750 rpm for 14 s (Omni tissue homogenizer TH115-K). The homogenized emulsion was diluted in 25 mL of 1% PVA (4 °C) and magnetically stirred under a fume hood for 3 h to allow evaporation of dichloromethane. PLGA microparticles were harvested by centrifugation at 5000 rpm for 10 min and washed three times in distilled water to remove excess PVA. DNA tracer was loaded into the microspheres during synthesis via cryopreparation, a technique that causes solidification of the initial emulsion containing DNA by lowering the temperature below its freezing point, using liquid nitrogen, before homogenization [19].

**Calcium alginate microparticles**: Calcium alginate microparticles were produced by adapting the water-in-oil emulsion method of Nograles et al. [20], and ionic gelation methods of Moradhaseli et al. [21] and Saraei et al. [22], using three different types of alginates. These included Protanal^®^ (viscosity 0.6–0.9 Pa·s in 1.25% H_2_O at 25 °C), low viscosity (LV, viscosity 4–12 Pa·s in 1% H_2_O at 25 °C), and medium viscosity (MV, viscosity ~14 Pa·s in 2% aqueous solution at 25 °C).

For the water-in-oil emulsion method [20], 625 μL of 0.1 M CaCl_2_ was mixed with 1.25 mL of 9:1 isoamyl alcohol: 3.5% (*w/v*) sodium alginate (MV, LV, and Protanal^®^) in the presence of 1% Span 80 by homogenization at 35,000 rpm for 10 s (Omni tissue homogenizer TH115-K). The emulsion was kept on ice for 30 min to stabilize and centrifuged at 3000 rpm for 3 min at 4 °C to collect particles.

The ionic gelation method of Moradhaseli et al. [21] was performed by mixing 0.5 mL of 1.8 mM CaCl_2_ and 2 mL of 0.05% PLL and adding 9.5 mL of 0.06% sodium alginate, and the suspension was stirred magnetically at 3000 rpm or homogenized (Omni tissue homogenizer TH115-K) at 8750 rpm for 30 min (Omni tissue homogenizer TH115-K). The suspension was centrifuged at 13,000 rpm for 30 min at 14 °C to harvest the CAM. Experiments were conducted with MV, LV, and Protanal^®^ alginates both in the presence and absence of 0.05% PLL.

Ionic gelation according to Saraei et al. [22] was undertaken by mixing 10 mL of 9 mM CaCl_2_ solution with 30 mL of 0.3% (*w/v*) LV sodium alginate solution on a stirrer (3000 rpm) or homogenizer (5000 rpm) at room temperature (Omni tissue homogenizer TH115-K). Following the addition of CaCl_2_, 500 μL of 0.1% PLL was added dropwise and stirred or homogenized at the same speed for 45 min at room temperature for particle formation. Calcium alginate microparticles were collected by centrifugation (13,000 rpm for 30 min at 14 °C).

CaCO_3_ microparticles without and with additives: CaCO_3_ microparticles were produced through precipitation [23] by adding 10 mL of 0.35 M Na_2_CO_3_ to an equal volume of 0.35 M CaCl_2_ under stirring at 27,500 rpm (Omni tissue homogenizer TH115-K). The solution was stirred for 30 s after the addition of Na_2_CO_3_, incubated at room temperature without stirring for 10 min, and centrifuged at 2500 rpm for 5 min to obtain CaCO_3_ microparticles. The particles were washed with deionized water. To produce particles containing biopolymer additives, CaCO_3_ was precipitated using equimolar solutions of 0.1 M Na_2_CO_3_ and CaCl_2_, in the presence of 0.1%, 0.3%, or 0.5% LV alginate.

### 2.2. Microparticle Visualization, DNA Loading, and Characterization

Microparticle synthesis was followed by morphology visualization, DNA loading, and characterization (surface charge and hydrophobicity). For morphology visualization, the microparticles were observed under a light microscope (DM6000B, Leica, Germany) for sphericity and size distribution. Biopolymer combinations and synthesis methods that yielded more uniform spherical microparticles were selected for DNA loading.

For DNA loading, the biopolymer microparticles were incubated overnight with 10^12^–10^16^ copies of the 352-base-pair-long, plasmid-free, double-stranded synthetic DNA tracer “K3”, under magnetic stirring [16]. The DNA loading efficiencies of different biopolymer microparticles were assessed by determining the copies of free DNA in the supernatant after the incubation period using quantitative polymerase chain reaction (qPCR). The following formula was used to determine the DNA loading efficiency:

(1) $$\mathrm{DNA}\;\mathrm{loading}\;\mathrm{efficiency}\;\left(\%\right)=\left[\frac{\left(\mathrm{initial}\;\mathrm{DNA}\;\mathrm{copies}\right)-\left(\mathrm{DNA}\;\mathrm{copies}\;\mathrm{in}\;\mathrm{the}\;\mathrm{supernatant}\right)}{\mathrm{initial}\;\mathrm{DNA}\;\mathrm{copies}}\right]\;\times \;100$$

Biopolymer microparticles with the highest DNA loading efficiency were characterized for their surface charge and hydrophobicity. To characterize surface charge, the zeta potential of the microparticles was measured in 2 mM NaCl (pH 6.5) using a Zetasizer Nano ZS (Malvern, UK). The hydrophobicity of the microparticles was evaluated by the microbial adhesion to hydrocarbon (MATH) assay [24]. The zeta potential and the relative hydrophobicity values of the microparticles and stationary-phase *L. pneumophila* cells [25] were compared prior to surface modification to understand the degree of hydrophobicity enhancement required.

### 2.3. Microparticle Surface Modification

The zeta potential and hydrophobicity of the selected microparticles were modified by assembling (1) PLGA or (2) polyelectrolytes PLL (positively charged) and PGA (negatively charged) on DNA-loaded microparticle surfaces using the layer-by-layer method, adapting methods of Luo et al. [26] and Powell et al. [27]. To assemble a PLGA layer, DNA-loaded microparticles were incubated in 2% (*w/v*) PLGA in dimethyl sulfoxide with continuous stirring for 1 h. Microparticles were then centrifuged at 4000 rpm for 3 min, washed with PLGA, and immediately transferred into 20 mL of 0.02% PVA solution and vortexed for 2 min. PLGA-coated microparticles were harvested by centrifugation (4000 rpm for 3 min) and washed with deionized water to remove excess PVA. PLL-PGA layer assembly methods for microparticle surfaces are discussed in detail in Ariyadasa et al. [16]. The zeta potential of the microparticles was measured to confirm the successful assembly of each layer. The MATH assay was used to evaluate the relative hydrophobicity of the surface-modified microparticles. Surface modifications were conducted until microparticle surfaces matched the zeta potential and surface hydrophobicity of stationary-phase *L. pneumophila* (−25.2 (±2.0) mV and 45.1 (±2.9)%, respectively [25]). These microparticles were further characterized for morphology using scanning electron microscopy (SEM) (JEOL 7000F FE-SEM, JEOL Ltd., Tokyo, Japan), porosity, pore volume using Brunauer–Emmett–Teller (BET) theory and the Barrer–Joyner–Halenda (BJH) model (Gemini VI 2385 surface area and pore size analyzer, micromeritics, Gwinnett County, GA, USA) [28], and particle concentration using qNano (IZON Science, Christchurch, New Zealand) using carboxylated polystyrene beads of 2000 nm diameter as calibration particles. To prepare samples for SEM, a drop of microparticle suspension was air-dried on a conductive carbon tab and sputter-coated with palladium for 180 s. Samples were observed at an operating voltage of 15.0 kV using the secondary electron and backscattering mode. Samples were degassed at 120 °C for 4 h prior to BJH analysis.

## 3. Results and Discussion

**Figure 1:**
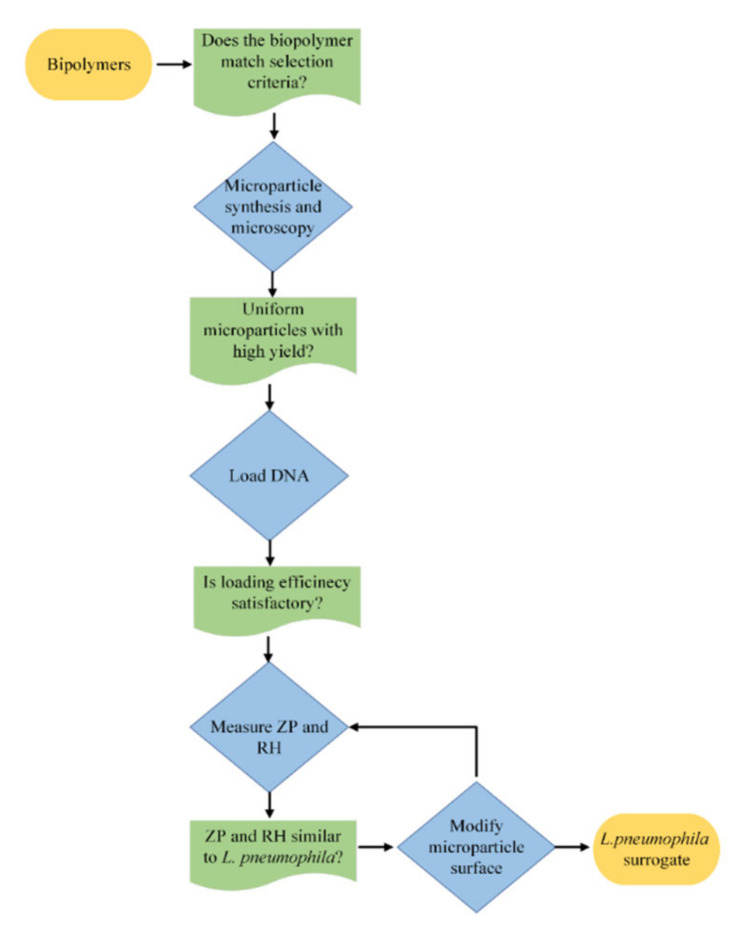
Stepwise approach to surrogate synthesis and modification. ZP: zeta potential; RH: relative hydrophobicity.

Biopolymers are known for their ability to form microparticles with varied structural and functional properties. In this study, we investigated different biopolymers for their ability to form microparticles with uniform size, controlled morphology, high DNA loading capacity, and tunable surface properties with the aim of developing a novel biopolymer surrogate for the waterborne pathogen *L. pneumophila*. A stepwise approach (Figure 1) was used, which included: (1) selection of biopolymer materials; (2) microparticle synthesis; (3) microparticle visualization, DNA loading, and characterization; (4) microparticle surface modification. Biopolymers were assessed in each stage to answer a “critical yes/no question” (represented in green, Figure 1) prior to conducting further experiments.

A literature survey was conducted to identify a range of candidate biopolymers suitable for microparticle synthesis, based on their zeta potential, material safety, and the existing knowledge. Biopolymers with a negative zeta potential were preferred, to mimic the cell surface charge of stationary-phase *L. pneumophila*. Therefore, inherently negatively charged biopolymers such as alginate, cellulose, κ-carrageenan, PLGA, carnauba wax, hyaluronic acid, and agarose were selected as opposed to positively charged polymers such as chitosan or poly-L-lysine. Proteins such as gelatine, collagen, whey, and casein were excluded due to the high thermal and pH dependence of their structures [29]. All the evaluated polymers were nonhazardous to aquatic life and contained no hazard/precautionary statements or label elements, according to the material safety data sheets. This ensured the possibility of future applications in eco-sensitive environments.

Microparticles were produced using the biopolymers identified in the literature, adapting protocols where necessary to achieve uniform microspheres that matched the size of stationary-phase *L. pneumophila* (1–2 μm in diameter; [25]). Table 1 presents a summary of morphologies, average diameters, and zeta potentials of the microparticles synthesized using the selected biopolymers. As indicated in Table 1, most biopolymers produced microspheres of varying sizes.

**Figure 2:**
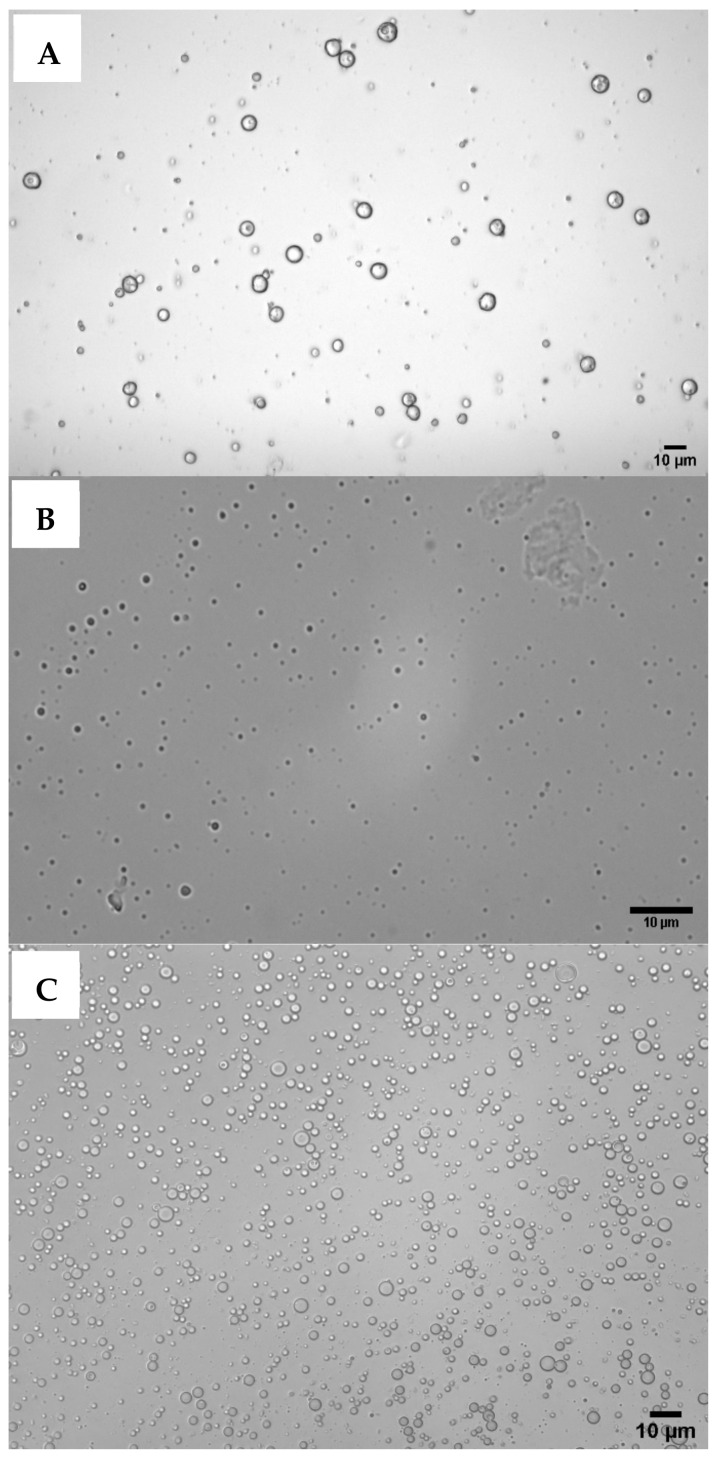
Light microscopy (40×) images of carnauba wax (**A**), κ-carrageenan (**B**), and PLGA (**C**) microspheres produced by adapting melt dispersion [17], emulsion [18], and water-in-oil-in-water double emulsion solvent evaporation [19] techniques, respectively.

Carnauba wax microparticles produced using our modified process parameters resulted in the formation of microspheres of 1–10 μm in diameter (Figure 2A), 4.9 ± 3.0 μm on average (d_90_/d_10_ = 4.77). This is significantly smaller in comparison with the average microsphere diameter of 231 μm reported in Milanovic et al. [17]. This is likely due to increased homogenization speed and time, resulting in higher disruptive mechanical forces breaking the interfacial tension. Only 12% of the microsphere population were between 1 and 2 μm in diameter and 53% between 2 and 5 μm. However, these microspheres were highly negatively charged (−49.2 ± 1.2 mV) and extremely hydrophobic (100%) compared to stationary-phase *L. pneumophila*.

κ-carrageenan microspheres with an average diameter of 1 ± 0.5 μm (d_90_/d_10_ = 3.16; Figure 2B) were obtained by adapting an oil emulsion technique [18] with a polysaccharide-to-oil phase ratio of 5% at 68 °C. While these conditions resulted in the formation of spherical structures, κ-carrageenan did not fully dissolve at 68 °C and additional centrifugation steps were required to separate the microsphere suspension from the undissolved polymer. Size analysis showed that 46% of κ-carrageenan gel microspheres were 1–2 μm in diameter. The zeta potential of these microspheres was −38.2 (±1.3) mV.

PLGA microspheres of uniform size and narrow size distribution (average diameter 2.0 ± 0.8 μm, d_90_/d_10_ = 2.7, Figure 2C) were produced by the water-in-oil-in-water double-emulsion solvent evaporation method [19]. Of these microspheres, 52.5% were 1–2 μm in diameter, whereas 47.5% were 2–4 μm. As this approach appeared promising, we also attempted loading tracer DNA during PLGA microsphere synthesis through cryopreparation. The DNA loading efficiency of PLGA microspheres was 70%. The zeta potential and hydrophobicity of these microspheres were −33.8 (±1.2) mV and 42.4 (±2.1)%, respectively.

**Figure 3:**
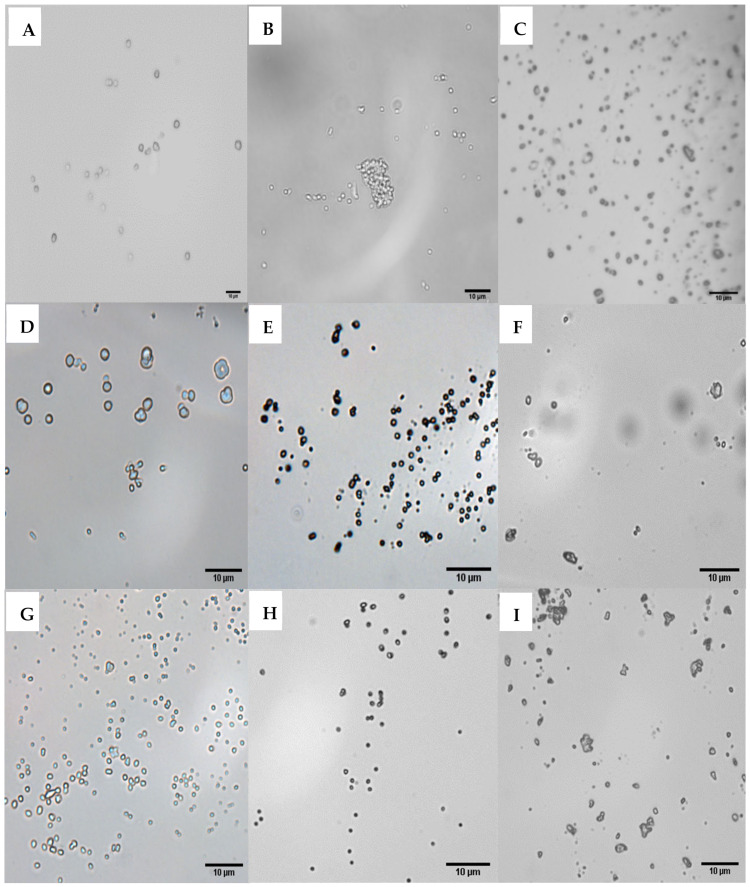
Light microscopy (40×) images of calcium alginate microparticles produced using water-in-oil (top row) and ionic gelation (middle and bottom rows, without and with poly-L-lysine, respectively) with different types of alginates. (**A**,**D**,**G**) were produced using medium-viscosity alginate, (**B**,**E**,**H**) using low-viscosity alginate, and (**C**,**F**,**I**) using Protanal^®^ alginate.

Alginate microspheres of various sizes and morphologies were produced using MV, LV, and Protanal^®^ alginate through the water-in-oil emulsion and ionic gelation methods (Figure 3). The three types of alginates were compared for the morphology and size of the microparticles they formed, as medium viscosity is known to influence microparticle properties [20,21,22]. All three types of alginates (at 3.5% *w/v*) produced microspheres via the water-in-oil emulsion method (Table 1), with MV alginate producing the largest spheres (MV > Protanal^®^ > LV, with average diameters of 2.3, 1.5, and 1.2 μm, respectively; Figure 3 top row) when the reaction mixtures were vortexed. Interestingly, homogenization of the reaction mixtures increased the microsphere diameter (MV > Protanal^®^ > LV; 15.5, 6.5, and 5.4 μm, respectively; data not shown in table). While this method was originally used to produce larger microspheres, 46.9 ± 3.1 μm in Nograles et al. [20], our results showed that an increase in the agitation time (from 20–30 s [16] to 3 min (present study)) resulted in microspheres with smaller diameters (Table 1).

Ionic gelation [21] of LV and MV alginate, both in the absence (Figure 3D,E) and presence of the cationic polyelectrolyte PLL (Figure 3G,H), resulted in the formation of microspheres, whereas Protanal^®^ alginate gave rise to irregular structures (Figure 3F,I). As shown in Table 1 and Figure 3 (middle and bottom rows), MV alginate produced larger microspheres (2.3 and 5.03 μm using Nograles et al.’s [20] and Moradhaseli et al.’s [21] methods, respectively) in comparison to LV alginate (1.2 and 3.1 μm using the same methods [20,21], respectively), and the size of the microspheres was more polydisperse (1 and 0.6, respectively) in the absence of PLL, compared to those formed in the presence of PLL (Table 1). The introduction of PLL into the reaction mixture not only reduced the microsphere size (0.76 μm and 0.3 μm using MV and LV alginate, respectively [21]) and polydispersity (0.5) but also caused a positive shift in their surface charge (30.7 and 38.9 mV, with MV and LV alginate, respectively). On the contrary, the ionic gelation technique of Saraei et al. [22] generated negatively charged LV alginate microspheres in the presence of PLL. Despite the difference in surface charge, these were similar in size (3.6 ± 2.0 μm) to the LV alginate microspheres synthesized following Moradhaseli et al.’s [21] method. However, none of these microspheres displayed a satisfactory DNA loading efficiency (41.2% and 13.8% for MV and LV microspheres produced using Moradhaseli et al. [21] and Saraei et al. [22], respectively).

**Figure 4:**
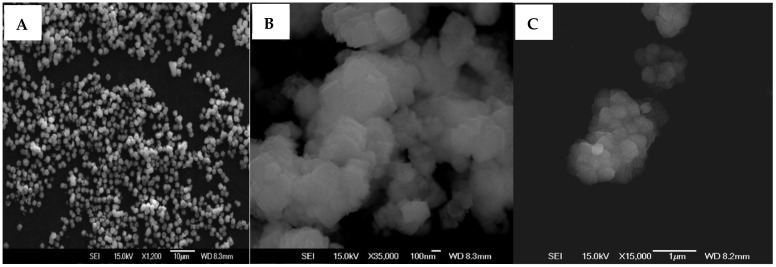
Scanning electron microscopy images of CaCO_3_ microparticles obtained without alginate (**A**), with 0.5% MV alginate (**B**), and with 0.1% LV alginate (**C**).

Alginate-CaCO_3_ microparticles were produced under homogenization, resulting in rod-shaped, uniform microparticles of ~1.3 μm in length and ~1 μm in width [16]. The introduction of 0.5% alginate into the coprecipitation reaction mixture containing equimolar solutions of Na_2_CO_3_ and CaCl_2_ (0.1 M each) unexpectedly resulted in the formation of microparticles with a unique rod-shaped morphology [16], whereas direct coprecipitation of the two salts (0.35 M) without alginate resulted in cuboid CaCO_3_ crystals (Figure 4A). It is known that the addition of negatively charged biopolymers such as alginate results in a deceleration of CaCO_3_ crystal formation, which may help enhance their effectiveness as delivery systems [30]. However, from the concentrations tested, the effect of alginate on crystal formation was only visible in the presence of 0.3 and 0.5% LV alginate, whereas 0.5% MV and 0.1% LV alginate resulted in CaCO_3_ aggregates with no defined morphology (Figure 4B). CaCO_3_ formed in the presence of 0.3% LV alginate consisted of flower-like microparticles (Figure 4C).

**Figure 5:**
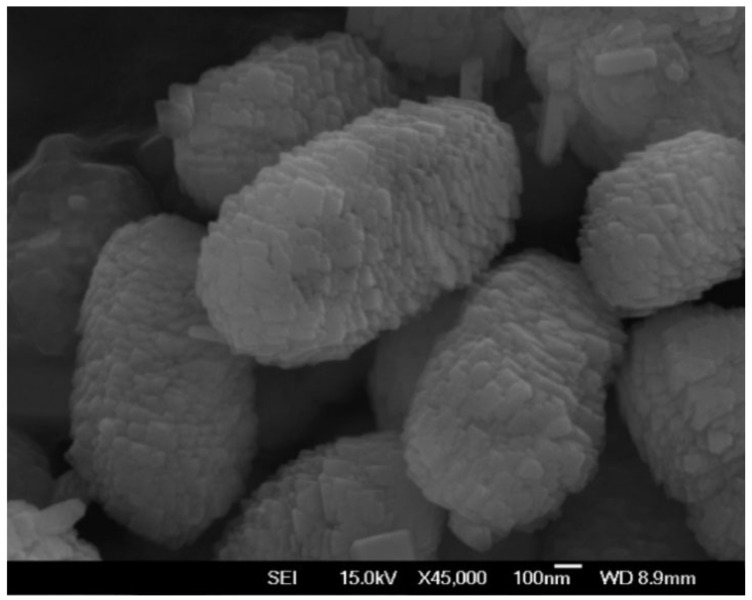
A high-resolution scanning electron microscopy image of rod-shaped alginate-CaCO_3_ microparticles.

Scanning electron microscopy indicated that the rod-shaped structures formed in the presence of 0.5% LV alginate comprised a distinctive, complex hierarchical superstructure made of self-assembled, uniform, axially oriented, nanoscale, “flake-like” plates of CaCO_3_ (Figure 5). The DNA loading efficiency, zeta potential, and hydrophobicity of the rod-shaped alginate-CaCO_3_ microparticles were 95.2 (±5.7)%, −26.1 (±0.4) mV, and 6.9 (±2.7)%, respectively [16].

The hydrophobicity of alginate-CaCO_3_ microparticles was significantly enhanced by surface modification. Alginate-CaCO_3_ microparticles modified with 2% PLGA-modified alginate-CaCO_3_ showed a hydrophobicity of 41.7 (±0.1)%, whereas 2% PLL-PGA modification resulted in a hydrophobicity of 37.9 (±0.5)%, compared to the 6.9 ± 2.7% hydrophobicity of unmodified particles. However, PLGA modification of DNA-loaded alginate-CaCO_3_ microparticles resulted in clump formation similar to those observed with PLGA. This may be due to the formation of aggregates due to PLGA and DNA interactions [19]. PLL-PGA modification did not alter the microparticle morphology. Alginate-CaCO_3_ microparticles were 1.0 ± 0.3 (d_90_/d_10_ = 2.1) μm and 1.3 ± 0.3 μm (d_90_/d_10_ = 2) in average diameter before and after PLL-PGA modification, respectively.

Of the biopolymers tested, only PLGA and alginate-CaCO_3_ produced size-controlled, uniform microparticles with high DNA loading efficiencies. Microspheres produced using carnauba wax, κ-carrageenan, and alginate were highly polydisperse and showed poor DNA loading efficiencies. PLGA microspheres closely resembled stationary-phase *L. pneumophila* in zeta potential, size, and hydrophobicity, whereas the surface properties of alginate-CaCO_3_ microparticles were easily modified using polyelectrolytes to obtain a zeta potential and hydrophobicity similar to *L. pneumophila*. Uniquely, in addition to size, zeta potential, and hydrophobicity, alginate-CaCO_3_ microparticles also closely mimicked the rod-shaped morphology of *L. pneumophila*, whereas PLGA microparticles were spherical.

Both PLGA and alginate-CaCO_3_ are extensively used as versatile carriers for controlled DNA delivery [30,31]. However, the PLGA microparticle synthesis process involves a volatile solvent, dichloromethane, which is toxic to humans and the environment. In addition, the shear forces generated by homogenization, sonication, and high interfacial tension may lead to DNA damage, although cryopreparation was used to minimize the impact of shear stress on DNA [19]. On the contrary, alginate-CaCO_3_ microparticles were synthesized by adapting a facile co-precipitation method using only nontoxic solutions. The high surface area and the mesoporous structure allowed efficient DNA loading through passive adsorption. As such, alginate-CaCO_3_ microparticles showed a higher DNA loading efficiency (95.2 ± 5.7%) compared to PLGA microspheres (70%). The PLL-PGA polyelectrolyte layer assembly may also act as a protective layer for DNA against environmental stresses in addition to enhancing microparticles’ hydrophobicity. Owing to these favorable characteristics, PLL-PGA-layered DNA-loaded alginate-CaCO_3_ microparticles were selected as the most suitable *L. pneumophila* surrogate for further validation experiments.

## 4. Conclusions

Various biopolymers and microparticle synthesis techniques were evaluated to identify a combination of biopolymers and microparticle preparation techniques to produce a novel, DNA-tracer-loaded biopolymer surrogate with a similar size, zeta potential, and hydrophobicity to stationary-phase *L. pneumophila*. The type of biopolymer, concentration, synthesis conditions, and method affected the morphology, size, zeta potential, hydrophobicity, and DNA loading efficiency of the microparticles. Our study showed that selecting the suitable combination of biopolymers and synthesis conditions helps to realize microparticles with morphologies and functions optimized for the selected application. The data gathered will assist others to develop microparticles with the desired properties, including mimics of other pathogenic microorganisms, which will unlock new safer approaches to environmental pathogen modeling and control.

PLL-PGA-modified DNA-loaded alginate-CaCO_3_ microparticles produced by adapting a facile co-precipitation method were selected as a suitable surrogate for *L. pneumophila* as they resulted in a high DNA loading efficiency and surface and morphological features that closely resembled the bacteria. In preliminary validation studies conducted using a laboratory-scale bioreactor device, this novel biopolymer surrogate displayed similar biofilm attachment/detachment kinetics as that of *L. pneumophila* [16], indicating its potential to mimic *L. pneumophila* mobility and persistence in water systems.

## Figures and Tables

**Table 1:** Summary of morphologies, average diameters, and zeta potentials of the microparticles synthesized using the selected parameters.

Biopolymer	Reference or Process Parameter	Microparticles			
		Morphology	Average Diameter (μm)	PDI	Zeta Potential (mV)
Carnuba wax	Milanovic et al. [18]	spherical	4.9 ± 3.0		(−49.2 ± 1.2)
K-carrageenan	Ellis and Jacquier [19]	spherical	1.0 ± 0.5		(−38.2 ± 1.3)
PLGA	Ando et al. [20]	spherical	2.0 ± 0.8		(−33.8 ± 1.7)
**Alginate only**					
medium viscosity	Nograles et al. [21], vortexed	spherical	2.3	1.0	−6.5
alginate	Nograles et al. [21], homogenized	spherical	15.5	1.0	−4.6
	Moradhaseli et al. [22], with PLL	spherical	0.8	0.5	30.7
	Moradhaseli et al. [22], without PLL	spherical	5.0	0.6	−14.0
Low viscosity	Nograles et al. [21], vortexed	spherical	1.2	0.7	−7.0
alginate	Nograles et al. [21], homogenized	spherical	5.4	0.4	−7.0
	Moradhaseli et al. [22], with PLL	spherical	0.3	0.5	38.9
	Moradhaseli et al. [22], without PLL	spherical	3.1	1.0	−16.7
	Saraei et al. [23]	spherical	3.6 ± 2.0		(−48.5 ± 2.3)
Protanal^®^	Nograles et al. [21], vortexed	spherical	1.5	0.9	−5.4
alginate	Nograles et al. [21], homogenized	spherical	6.5	1.0	−5.3
	Moradhaseli et al. [22], with PLL	mostly irregular	3.8	0.8	30.1
	Moradhaseli et al. [22], without PLL	mostly irregular	4.1	0.1	−13.3
**CaCO_3_ only**					
CaCO_3_ without additives	Wang et al. [24]	cuboid	ND		(−25.9 ± 0.4)
**Alginate-CaCO_3_ composite**					
Medium-viscosity alginate**-CaCO_3_**	0.5% MV alginate + 0.1 M CaCO_3_	aggregates with no defined morphology	ND		(−31.1 ± 0.3)
Low-viscosity alginate**-CaCO_3_**	0.1% MV alginate + 0.1 M CaCO_3_	aggregates with no defined morphology	ND		(−24.6 ± 0.4)
	0.3% MV alginate + 0.1 M CaCO_3_	flower-like formations	ND		(−33.4 ± 1.0)
	0.5% MV alginate + 0.1 M CaCO_3_	rod-shaped	1.0 ± 0.3		(−26.1 ± 0.4)
PLGA-modified Alginate-CaCO_3_	(0.5% MV alginate + 0.1 M CaCO_3_) + 2% PLGA	rod-shaped	1.9	0.6	(−13.9 ± 0.4)
** PLL-PGA- modified Alginate-CaCO**_3_**	Ariyadasa et al. [17]	rod-shaped	1.3 ± 0.3		(−21.7 ± 0.9)
Stationary-phase *L. pneumophila*	Ariyadasa et al. [26]	rod shaped	1.4 ± 0.3 μm long; 0.32 ± 0.03 μm wide		(−27. 2 ± 0.1)

PDI = polydispersity index, ND = Not Determined, PLGA = poly (lactic-co-glycolic acid), PLL = poly-L-Lysine, MV = medium viscosity, ** *L. pneumophila* surrogate.

## Data Availability

Not applicable.
